# Nutritional and Rheological Characterization of an Infant Flour Based on Parboiled Rice (*Oryza sativa*), Spirulina (*Spirulina platensis*), and Cashew Nut (*Anacardium occidentale*)

**DOI:** 10.1155/2022/3784317

**Published:** 2022-09-02

**Authors:** Love Mouka Mahouli, Bruno Fotso Saah, Hygride Dongmo, Justine Odelonne Kenfack, Bilkissou Njapndounke, Marie Madeleine Nanga Ndjang, Mathilde Julie Klang

**Affiliations:** ^1^Research Unit of Biochemistry of Medicinal Plants, Food Sciences and Nutrition, University of Dschang, P.O. Box 67, Dschang, Cameroon; ^2^Centre for Natural Products Research, Department of Chemical Science, University of Johannesburg, P.O. Box 17011, Doorfontein Campus, Gauteng 2028, South Africa

## Abstract

Protein-energy malnutrition and mineral deficiencies in children under five years are major problems in developing countries. The present study was thus carried out with the aim of proposing a weaning flour based on parboiled rice, spirulina, and cashew nut that meets the nutritional needs of children aged 06 to 24 months. To achieve this, the mixture design approach (a 9-point augmented simplex-centroid design) was used to obtain optimal blend of flour. The responses evaluated where proteins, lipids, carbohydrates, minerals (iron, calcium, phosphorus, potassium, sodium, magnesium, and zinc), carotenoids, fibers, and ash content. The rheological analyses (rapid viscosity analysis) were carried out on the optimal flour. It results that the optimal proportion of parboiled rice, spirulina, and cashew nut was, respectively, 85.80, 5.96, and 8.23. These conditions result in a protein, lipids, carbohydrates, carotenoids, ash, fibers, calcium, iron, magnesium, and zinc content of 14.15%, 9.04%, 68.46%, 8.2 mg, 3% ash, 4.98%, 510 mg, 16 mg, 65 mg, and 5 mg, respectively, and an energy value of 411.8 kcal. These optimate conditions resulted also in a peak viscosity of 60 cP and a final viscosity of 86 cP which are lower than the established value (1000 cP). Thus, the weaning flour based on parboiled rice, spirulina, and cashew nut obtained in these optimate conditions can meet the nutritional needs of children aged 06 to 24 months and is therefore an efficient weaning food to fight against child malnutrition.

## 1. Introduction

Malnutrition is a major public health problem that affects 821 million people worldwide. In sub-Saharan Africa, 22.8% of children under the age of 5 suffer from this malnutrition [1]. In Chad, 31.9% or nearly 1/3 of children fewer than 5 suffer from chronic malnutrition. The most affected regions are Lake Chad (48.2%), Kanem (37.1%), Mayo-Kebbi (33.9%), and Logone (28.5%) [2]. Kone et al. [3] report that the main causes of this malnutrition are deficiencies in energy, proteins, and certain micronutrients such as iron, calcium, zinc, and beta-carotene, which regulate a range of metabolic processes in humans. Indeed, after the first six months of life, breast milk becomes qualitatively and quantitatively insufficient to cover the growing nutritional needs of the child [4]. In this period, the child needs a special diet providing sufficient energy, proteins, vitamins, and minerals [3]. Therefore, it is important to develop foods that meet these nutritional needs. Though imported supplementary foods can meet these needs, their high cost makes them inaccessible to low-income populations [5]. This is the reason while, in Africa, supplementary foods are most often made from available cereals and tubers with or without sugar [6]. Although these cereals and tubers are very rich in carbohydrates, nevertheless, they are poor in proteins and some micronutrients [7]. However, besides these carbohydrate-rich matrices (starch), like rice, there is a number of foods in Chad that are rich in proteins, lipids, and certain minerals and vitamins. These include cashew nut and spirulina, which are available and less expensive. Cashew nut is rich in lipids (48.3 g/100 g), proteins (21.3 g/100 g), and minerals such as potassium (700 mg/100 g) and calcium (200 mg/100 g) [8]. As for spirulina, it is a microalgae very rich in proteins (60-70 g/100 g) and carotenoids (440 mg/100 g) including 140 mg/100 g of beta-carotenes, calcium (120 mg/100 g), and other minerals such as iron, potassium, magnesium, phosphorus, and zinc [9].Thus, appropriate weaning flours for the second age (06-24 months) must be formulated with locally available products which should be composed of 15% protein, 7-10% fat, 68% carbohydrates, 2-5% ash, and 2-5% fiber with an energy value of about 400 kcal per 100 g of flour and a flow rate of 120-160 mm/30 s [10]. It is in this view that [5] showed that the addition of soybean flour (25%) and Moringa (5%) powder increased the proteins, energy, and minerals density of cassava flour (70%). In the same way, [3] showed that the incorporation of cashew nut flour (10%, 15%, and 20%) into attiéké flour significantly improved its nutritional value. However, all of the above works have limitations in the sense that some did not use mathematical tools such as mixture design to reduce the number of samples and determined the adequate proportions of each component of the mixture. In addition, some of the matrices used such as soybean, potato, and attiéké are rare in Chad; meanwhile, rice, spirulina, and cashew nuts are available in abundance and can be used to formulate weaning flour. Thus, the present work was initiated with the objective of formulating a weaning flour based on parboiled rice flour, spirulina, and cashew nut, which meet the nutritional needs of children aged 06-24 months.

## 2. Materials and Methods

### 2.1. Plant Material

The paddy rice (*Nerica L56*) was purchased from a farmer in Koutaba, the spirulina from Bol market, and the cashew nut was collected in Garoua. The samples thus collected were forwarded to the laboratory and proceeded into flour and powders for weaning food production.

### 2.2. Production of Flour and Powders

#### 2.2.1. Parboiled Rice Flour

The paddy rice previously cleaned (winnowed and sorted, then washed) was soaked in hot water at 65°C for 6 hours and drained. It was then steamed for 40 min, dried in the shade for one hour, and parboiled for 18 h at 45°C. Once parboiled, the paddy was husked, winnowed, and then milled to obtain flour (300 *μ*m).

#### 2.2.2. Spirulina Powder

The spirulina collected in Lake Chad basin (latitude: 13°27′31^″^N, longitude: 14°42′53^″^E, and altitude: 301 m) was filtered through a very fine sieve and washed to remove impurities. It was then pressed in order to reduce the quantity of water, then spread, and dried at 45°C during 24 hours. Once dried, it was crushed and sifted to obtain a powder of 300 *μ*m.

#### 2.2.3. Cashew Nut Flour

The collected cashew nuts were cleaned and steamed for 45 min in order to obtain kernels. The wrapped kernel was dried (45°C, 12 h) to facilitate the dehulling which consists in removing the envelope which surrounded the almond. The almond was then dried, crushed, and sieved at 300 *μ*m.

### 2.3. Formulation of the Optimal Flour

A 9-point augmented simplex-centroid design was applied to obtain the optimal flour. The augmented centered mixing designs explore the entire area of interest of a mixing design and conduct mixing experiments perfectly. Designing was done using Minitab 19.1.0, and the experiments were carried out in triplicate.

#### 2.3.1. Choice of Components, Domain, and Experimental Matrix

Three factors were identified: the amount of parboiled rice flour (PRF), the amount of spirulina powder (SP), and the amount of cashew nut flour (CNF). The responses were proteins, lipids, carbohydrates, minerals (iron, calcium, phosphorus, potassium, sodium, magnesium, and zinc), carotenoids, fibers, and ash content. The choice of experimental domains was made based on data from the literature review and the chemical composition of each matrix. After choosing the intervals of the different factors, we proceeded to the determination of the number of experiments to be carried according to a three-factor mixture design (Table 1).

#### 2.3.2. Proposed Model

The postulated model can be either linear or polynomial of second-degree where the responses are coded by (*Y*). The fundamental constraint of the mixtures makes the constant disappear, and the second-degree terms reduce to rectangles. The linear mathematical model of the formulated flour was as follows:

(1) $$\begin{array}{c} Y=\beta_{1}\text{ PRF}+\beta_{2}\text{ SP}+\beta_{3}\text{ CNF}. \end{array}$$

The second-degree polynomial mathematical model was as follows:

(2) $$\begin{array}{c} Y=\beta_{1}\text{ PRF}+\beta_{2}\text{ SP}+\beta_{3}\text{ CNF}+\beta_{12}\text{ PRF}*\text{SP}+\beta 1_{3}\text{ PRF}*\text{CNF}+\beta 2_{3}\text{ SP}*\text{CNF}, \end{array}$$

where *Y* is the expected response, *β*_1_, *β*_2_, and *β*_3_ are linear coefficients, *β*_12_, *β*_13_, and *β*_23_ are interaction coefficients, and PRF, SP, CNF, PRF^∗^SP, PRF^∗^CNF, and SP^∗^CNF are levels of independent variables.

#### 2.3.3. Models' Validation

The validation and strength of an experimental mathematical model require the determination of a number of parameters such as the determination coefficient (*R*^2^) which must be above 75%, the Absolute Mean Deviation Analysis (AMDA) which must be equal to 0, and the bias factor (Bf) which must be between 0.75 and 1.25 [11].

### 2.4. Assessment of Responses

#### 2.4.1. Nutritional Composition of the Different Flours and the Optimal Flour

The proteins content was determined by the Kjeldahl method [12], where the protein content is deduced from the nitrogen content by the following formula: %Proteins = %*N* × conversion factor; with a conversion factor equal to 6.25; lipid content by the Soxhlet method [12]; and total carbohydrates by difference of dry weight where the weight of ash, proteins, and lipids are differentiated [12] according to the following formula: %Carbohydrates = DM–(%Proteins + %Lipids + %ash + %fibers). The ash content was in accordance with the method [12]. Crude fibers were in accordance with [12], and the energy value was calculated using Atwater's specific coefficients for proteins, lipids, and carbohydrates [13], based on the following formula: EV = (4 × (%carbohydrates)) + (9 × (%lipids)) + (4 × (%proteins)).The carotenoids were determined by the averaging method described by [14].

#### 2.4.2. Determination of Rheological Characteristics

The pasting properties of optimized flour were characterized by Rapid Viscosity Analysis (RVA). Starch dispersed in water with constant agitation was subjected to a Brabender viscoamylograph which records the evolution of the consistency of the paste during different phases of heating, temperature maintenance, or cooling. The viscoamylograph reflecting the transformations undergone by the starch granules as a function of time during heating shows the evolution of its consistency. To achieve this, 3 g of flour was mixed in 25 ml of distilled water. The resulting suspension was subjected to a progressive heating at the rate of 6°C/min from 50 to 95°C. The constant temperature of 95°C was maintained for 5 min, followed by a cooling of the suspension at the rate of -12°C/min. A new constant 57°C temperature step was maintained for 2 min. Temperatures of the aqueous starch suspension and recorded viscosities (in centipoise or cP) in real time were deduced from the starch weighing parameters. The following viscometric parameters were measured in this work:

- Pasting temperature (PT) (°C)
- Peak viscosity (PV) (cP)
- Holding viscosity (HV) (cP)
- Breakdown (B) (cP)
- Final Viscosity (FV) (cP)
- Setback (S) (cP)

### 2.5. Statistical Analysis

Graphical representations of the isoresponse curves of the postulated models were performed using Minitab version 19.1.0 software. Data were analyzed with Excel 2013 and Minitab version 19.1.0 software and statistical analyses using SPSS version 22.0 software. The comparison of means was performed by the ANOVA test to classify the treatments in case there was a significant difference. The difference between two data is significant when the actual significance level is below 5%.

## 3. Results and Discussion

### 3.1. Presentation of the Different Responses

Table 2 shows the different factors and responses of each mixture obtained in the formulation of weaning flour base on parboiled rice flour, spirulina, and cashew nut. From this table, it is observed that the responses vary according to the incorporation rate of different components in each test. In these 09 tests, an increase in carbohydrate and lipid content is observed, which vary from 60.64 to 70.43% and 8.10 to 10.51%, respectively, according to the incorporation rate of FRP in each mixture. Indeed, rice is considered an important source of carbohydrates [4], and cashew nuts are a good source of lipids: 40-57 g/100 g [3]. For protein, the content increases from 14.06 to 22.18%, and this is mainly due to the incorporation of PS which is known to be rich in protein: 60-70 g/100 g and minerals and carotenoids: 140 mg/100 g [9]. In the same way, the content of ash (2.2-4.9%), iron (13.8-21.56 mg/100 g), calcium (538.50-699.80 mg/100 g), zinc (3.5-5.8 mg/100 g), and carotenoids (8.63-24.04 mg/100 mg) varies with the rate of PS in the different mixtures.

### 3.2. Prediction of Mathematical Models

The following regression equations were obtained after analysis of the different responses (P, L, C, A, TF, Na, Ca, Zn, Fe, Mg, P, K and carotenoids). These responses (*Y*) are expressed as a function of PRF, SP, and CNF which represent the effects of parboiled rice, spirulina, and cashew nut on the response, respectively. The higher the coefficient of these factors, the more the factor influences the response. PRF∗SP represents the interactions of parboiled rice and spirulina on the response, and PRF∗CNF represents the interactions of parboiled rice and cashew nut on the response. The SP∗CNF terms cannot be estimated; so, they were removed from the equation. It shows that cashew nut flour is the factor that contributes the most to the equations for proteins, carbohydrates, ash, iron, calcium, zinc, magnesium, phosphorus, and carotenoids. The factor that influences the most potassium, sodium, fibers, and lipids is spirulina. Cashew nut flour contains various factors such as tannins, phytic acid, amylase inhibitors, and trypsin inhibitors that limit the absorption of carbohydrates, proteins, and minerals [15].

Here are the following factors:

(i) Proteins:
  (3) $$\begin{array}{c} Y_{1}=0.11\text{ PRF}+0.53\text{ SP}+0.71\text{ CNF}+0.00\text{ PRF}*\text{SP}-0.00\text{ PRF}*\text{CNF}. \end{array}$$
(ii) Lipids:
  (4) $$\begin{array}{c} Y_{2}=0.05\text{ PRF}+0.69\text{ SP}-0.54\text{ CNF}-0.00\text{ PRF}*\text{SP}+0.01\text{ PRF}*\text{CNF}. \end{array}$$
(iii) Carbohydrates:
  (5) $$\begin{array}{c} Y_{3}=0.77\text{ PRF}-1.38\text{ SP}+2.82\text{ CNF}+0.01\text{ PRF}*\text{SP}-0.03\text{ PRF}*\text{CNF}. \end{array}$$
(iv) Ash:
  (6) $$\begin{array}{c} Y_{4}=-0.00\text{ PRF}+0.92\text{ SP}-1.49\text{ CNF}-0.00\text{ PRF}*\text{SP}+0.02\text{ PRF}*\text{CNF}. \end{array}$$
(v) Fibers:
  (7) $$\begin{array}{c} Y_{5}=0.05\text{ PRF}+0.51\text{ SP}+0.21\text{ CNF}+0.00\text{ PRF}*\text{SP}-0.00\text{ PRF}*\text{CNF}. \end{array}$$
(vi) Iron:
  (8) $$\begin{array}{c} Y_{6}=0.09\text{ PRF}-0.23\text{ SP}+0.66\text{ CNF}+0.01\text{ PRF}*\text{SP}-0.00\text{ PRF}*\text{CNF}. \end{array}$$
(vii) Calcium:
  (9) $$\begin{array}{c} Y_{7}=1.09\text{ PRF}-160.59\text{ SP}+46.08\text{ CNF}+2.40\text{ PRF}*\text{SP}-0.21\text{ PRF}*\text{CNF}. \end{array}$$
(viii) Sodium:
  (10) $$\begin{array}{c} Y_{8}=-0.13\text{ PRF}-11.48\text{ SP}-1.40\text{ CNF}+0.22\text{ PRF}*\text{SP}+0.12\text{ PRF}*\text{CNF}. \end{array}$$
(ix) Zinc:
  (11) $$\begin{array}{c} Y_{9}=0.01\text{ PRF}+0.22\text{ SP}-0.38\text{ CNF}-0.00\text{ PRF}*\text{SP}+0.00\text{ PRF}*\text{CNF}. \end{array}$$
(x) Magnesium:
  (12) $$\begin{array}{c} Y_{10}=-0.89\text{ PRF}+22.61\text{ SP}-93.39\text{ CNF}-0.21\text{ PRF}*\text{SP}+1.25\text{ PRF}*\text{CNF}. \end{array}$$
(xi) Potassium
  (13) $$\begin{array}{c} Y_{11}=5.14\text{ PRF}+26.47\text{ SP}-24.23\text{ CNF}-0.19\text{ PRF}*\text{SP}+0.38\text{ PRF}*\text{CNF}. \end{array}$$
(xii) Phosphorus
  (14) $$\begin{array}{c} Y_{12}=0.38\text{ PRF}+50.04\text{ SP}+59.20\text{ CNF}+0.82\text{ PRF}*\text{SP}-0.52\text{ PRF}*\text{CNF}. \end{array}$$
(xiii) Carotenoids:
  (15) $$\begin{array}{c} {\text{Y}}_{13}=0.16\text{ PRF}+3.67\text{ SP}+3.83\text{ CNF}-0.04\text{ PRF}*\text{SP}-0.05\text{ PRF}*\text{CNF}. \end{array}$$

### 3.3. Model Validation

Table 3 presents the coefficient of determination (*R*^2^), the AAMD (absolute analysis of the mean deviation), and the Bf (bias factor) obtained for the analysis of the different responses. It appears that the *R*^2^ of the different responses is greater than 75%, the AADM is equal to 0, and the Bf is between 0.75 and 1.25. Indeed, when these three coefficients respected standard values, we consider that there is an adequacy between the experimental responses and the predicted responses given by the model [16].

### 3.4. Optimal Conditions for Formulated Infant Flour

**Figure 1:**
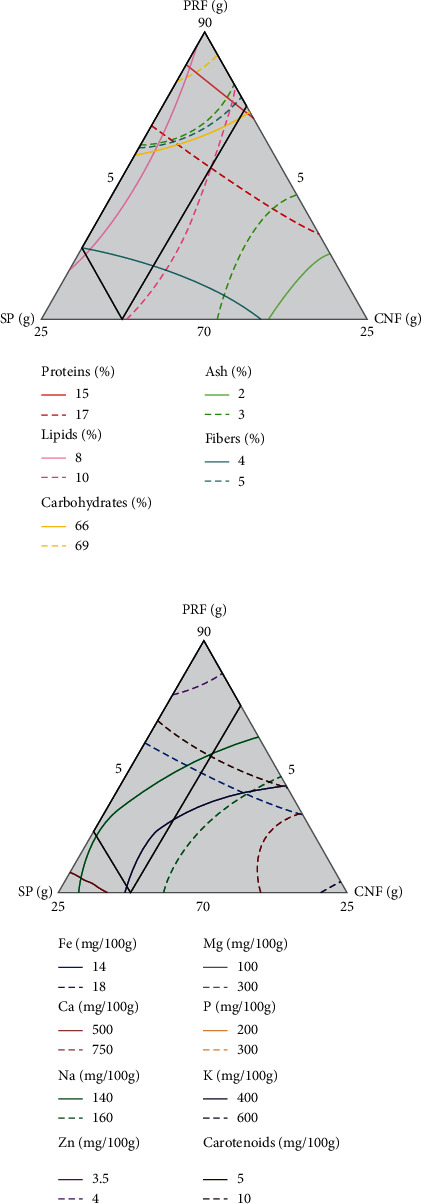
Overlaid contour plots for optimum composition for weaning flour.

After the analysis of the different responses, a compromise was made between the different optimal conditions obtained from the responses obtain by the different tests. The rectangle of Figure 1 indicates the optimum area (compromise zone) of the formulation that should be used to develop weaning flour with the best chemical composition which can serve the expected purpose. This optimum area includes all the optimum points of proteins, lipids, carbohydrates, fibers, ash, iron, calcium, sodium, phosphorus, potassium, magnesium, zinc, and carotenoids. In optimization process, the superimposition of contour plot regions of interest considered 15 < proteins < 17, 8 < lipids < 10, 66 < carbohydrates < 69, 2 < ash < 3, 4 < fibers < 5, 14 < iron < 18, 500 < calcium < 750, 140 < sodium < 160, 200 < phosphorus < 300, 400 < potassium < 600, 100 < magnesium < 300, 3.5 < zinc < 4, and 5 < carotenoids < 10. All this optimum result of chemical composition was the best for 6-24 months old babies according to the nutritional guidelines [10]. Therefore, to formulate a weaning food of good nutritional value based on parboiled rice flour, spirulina, and cashew nut, 85.80%, 5.96%, and 8.23% of these flours must be taken, respectively.  Table 4 presents the predicted optimal responses, the experimental optimal responses, and desirability. According to this table, the optimal experimental responses represent the nutritional composition of our chosen flour. Moreover, we note no significant difference (*p* < 0.05) between the responses given by the model and those obtained in the laboratory, and the desirability varies from 0.92 to 1. Thus, our optimal proportions of parboiled rice flour, spirulina, and cashew nut flour are validated.

### 3.5. Characterization of the Optimal Infant Flour

#### 3.5.1. Nutritional Composition of the Optimal Infant Flour

Table 5 presents the nutritional composition of the flour formulated with 85.80% PRF, 5.96% SP, and 8.23% CNF. It emerges that the protein content obtained (14.02%) respects the standards of [17] which are 12-15% proteins. This high protein content of the formulated flour is advantageous for the child growth as proteins would have a positive impact on tissue repair and muscle building [18]. As for the lipid content of a weaning food according to [17], it should be between 7 and 10%. Thus, the lipid content (9.04%) of the formulation respects the standard of [17]. Indeed, these lipids produce 02 times more energy to the body. Carbohydrates are macromolecules that also play an energy role in the body. Their content in the optimal flour is 68.48%, mainly influenced by the quantity of parboiled rice. This value is in respect with the value (68%) established by the [17] for infant flours for children aged 06-24 months. The ash content for its part is an indicator of flour purity. In the optimal flour, it is 3%, and it is influenced by the amount of cashew nut and spirulina incorporated. This value meets the standards of [17] which are 2-5%. According to [17], the fiber content of infant flours should not exceed 5 g per 100 g of product; it should be between 2 and 5 g. The optimal flour has a fiber content of 4.98% which is in the recommended range. This high fiber content of the formulated flour is mostly influenced by the addition of spirulina in the formulation. It is advantageous in the sense that it could regulate the intestinal transit and capture part of the lipids and carbohydrates to allow the regulation of the blood sugar level thus avoid the excess of cholesterol [19].

The mineral content is mainly influenced by the amount of spirulina and cashew nut in the formulated flour. The optimal values obtained for iron, calcium, sodium, zinc, magnesium, potassium, and phosphorus are, respectively, 16 mg, 510 mg, 123 mg, 5 mg, 65 mg, 581,8 mg, and 282,5 mg, per 100 g of dry matter. The amounts of iron, potassium, phosphorus, and sodium are in accordance with the recommended values [17]. However, the levels of magnesium, calcium, and zinc are higher than the minimum values recommended by [17]. The presence of all these minerals in the formulated flour is benefic, as they are involved in the development of the child's organism. It can also limit the occurrence of nutritional deficiencies. Iron is involved in the constitution of hemoglobin, myoglobin, and of many enzymes, and it is also essential for a large number of metabolic reactions [4]. Calcium and phosphorus ensure bone rigidity and promote child growth during the weaning period. Zinc, along with iron, is the most concentrated mineral in the brain. It is therefore essential to cover the zinc needs of infants when brain growth is still important. Zinc is also involved in immunity because it reduces the incidence and severity of diarrhea in children. Magnesium is necessary for biochemical reactions in the body, maintaining muscles, improving nerve function, maintaining heart rate, and regulating blood sugar [4]. Carotenoids are generally tetraterpenoid phytochemicals that cannot be synthesized by humans. Some such as *β*-carotene can be metabolized into vitamin A [14]. Carotenoid content of the optimal flour is 8.2 mg dry matter which is more influenced by the amount of spirulina. Carotenoids are an important source of vitamin A (*β*-carotene, *β*-cryptoxanthin, *α*-carotene), as they contribute to their intake and also have a great antioxidant capacity. They can inhibit the propagation of lipid peroxidation and decrease oxidative damage to DNA, thus protecting against cancer. They are also very important as immunomodulators and in cellular communication [20].

Energy value of the optimized weaning flour is within the value recommended by [10] for children of 06-24 months age.

#### 3.5.2. Rheological Properties of the Optimal Infant Flour

**Figure 2:**
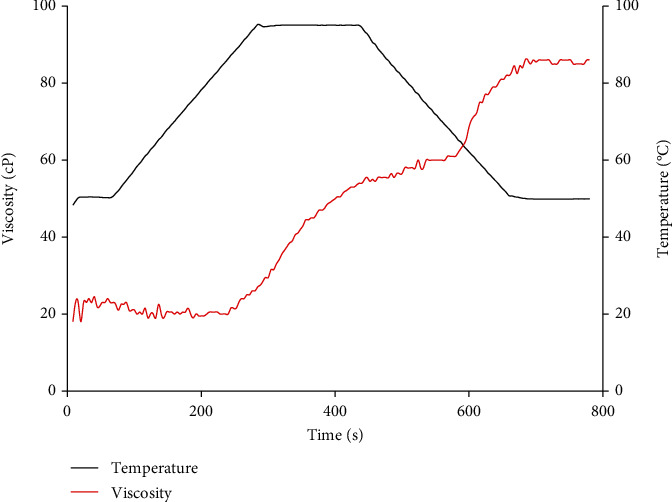
Viscosity profile of formulated flour under heating and cooling.

Figure 2 shows the rheological profile of the optimum flour, and Table 6 presents the pasting properties given calculated on the basis of this graph. It can be seen that the starch granules of the optimal flour start to swell at a pasting temperature, which indicates the minimum energy required to initiate the rapid water uptake and swelling of the starch granules which result in an increased of the viscosity. This pasting temperature obtained is due to the granules size composing the flour the optimal flour and the disorganization and denaturation of the starch granules in the parboiled rice flour [21]. In addition to this, the high presence of proteins 14.02% and lipids (9.04%) complexes the starch and prevents it from swelling. The pasting temperature obtained in this work is higher than that obtained by [22]. This difference may be due to the fact that their formulations incorporated sprouted millet flour, and the latter being rich in amylases will decrease the starch content of the fava and thus its pasting temperature.

Subsequently, an increase in viscosity is observed with increasing temperature until the maximum peak (peak viscosity). This peak viscosity indicates the thickening and water holding capacity of the starch [23]. Thus, this observed increase is reflected in the maximum swelling of starch granules, as well as their water binding capacity.

The peak viscosity of the optimal flour obtained can be further explained by its low amylose content (19.75 g), the particle size of the starch source (parboiled rice flour), and amylose-proteins and amylose-lipid interactions [24]. This is therefore in agreement with the work of [25], which showed that flours that have high starch content will have high swelling power and high peak viscosity. The viscosity remains increasing and merges to the holding viscosity between 60 and 75°C. The breakdown of starch thus becomes zero in the formulated flour and leads to a high breakdown ratio. This is because the shear strength reflects the viscosity of the starch at 95°C until a stable leg is formed and thus provides information on the stiffness of the starch granules.

Breakdown and holding viscosity refer to the degree to which the viscosity of starches drops after gelatinization [20]. It can be seen from Table 6 that holding viscosity and breakdown of optimized flour are zero and thus lead to a high resistance to shear stress. This is explained by pregelatinization of rice starch during parboiling.

Then, a gradual increase in viscosity to final viscosity is observed. This is reflected in the ability of the starch to retrograde upon lowering the temperature due to molecular rearrangement and the formation of amylose-lipid complexes [26]. Thus, the final viscosity of the formulated flour shows a low retrograde capacity of the formulated flour, which could be explained by the bursting of the starch granules upon water absorption and the size of the starch granule residues. The final viscosity thus obtained for our flour is lower than that established by the standard (1000 cP), hence the importance of this flour in the fight against malnutrition.

The difference between the final viscosity and the holding viscosity represents the ability of the starch to retrograde. According to [27], this is an indicator of the rearrangement of the amylose molecules during cooling by the formation of hydrogen bonds between the hydroxyl groups released during gelatinization.

The setback of the optimal formulated flour is lower than that obtained by [28]. This difference can be explained by the chemical composition of the formulated flour, mainly amylose, proteins, and lipid content [27]. The low setback of the optimized flour can also be due to the decrease in the number of native granules caused by partial gelatinization during the soaking and steaming operations of parboiling rice [29].

## 4. Conclusion

The analyses carried out show that the different samples used for the formulation through the mixing plan allowed to find the optimal conditions. Thus, to have a good weaning food of nutritional quality meeting the standards, 85.80% of parboiled rice flour, 5.96% of spirulina, and 8.23% of cashew nut are required. The final viscosity obtained for our flour is lower than that established by the standard and therefor meets the nutritional needs of children in fewer meals, thus allowing a more effective fight against protein-energy malnutrition and hidden hunger. In view of these results, the formulation of a weaning food based on parboiled rice flour, spirulina, and cashew nut enables us to have a weaning food of good nutritional value and acceptable rheological properties. This study also provides alternatives to the weaning foods in Chadian market as well as in the subregion. The addition of spirulina and cashew nut enriched the rice in the required contents in proteins, lipids, minerals, and carotenoids.

## Figures and Tables

**Table 1:** Experimental matrix.

	Coded variables			Real variables		
Tests	PRF	SP	CNF	PRF (g)	SP (g)	CNF (g)
1	-0.5	0.5	0.5	75.0	16.25	8.75
2	0.5	-1	1	85.0	5.00	10.00
3	-0.5	1	-1	75.0	20.00	5.00
4	0	0	0	80.0	12.50	7.50
5	-0.25	0.5	-0.5	77.5	16.25	6.25
6	1	1	1	70.0	20.00	10.00
7	0.5	-0.5	-0.5	85.0	8.75	6.25
8	0.25	-0.5	0.5	82.5	8.75	8.75
9	1	-1	-1	90.0	5.00	5.00

PRF: parboiled rice flour; SP: spirulina powder; CNF: cashew nut flour.

**(a):** 

	Factors			Responses				
Tests	PRF (g)	SP (g)	CNF (g)	P (g)	L (g)	C (g)	A%	TF%
1	75	16.25	8.75	19.87 ± 1.32	9.20 ± 0.76	61.32 ± 9.45	4 ± 0.34	4.15 ± 0.33
2	85	5	10	14.86 ± 2.24	10.51 ± 0.45	66.21 ± 6.45	3.2 ± 0.75	4.98 ± 0.56
3	75	20	5	20.92 ± 1.02	7.84 ± 0.62	60.64 ± 1.39	4.71 ± 0.78	3.98 ± 0.12
4	80	12.5	7.5	18.12 ± 2.32	8.75 ± 0.22	64.46 ± 6.83	3.4 ± 0.43	4.85 ± 0.18
5	77.5	16.25	6.25	19.47 ± 1.96	8.08 ± 0.33	63.46 ± 5.33	3.91 ± 0.33	4.51 ± 0.11
6	70	20	10	22.18 ± 3.67	9.90 ± 0.92	57.75 ± 0.34	4.9 ± 0.34	3.28 ± 0.23
7	85	8.75	6.25	15.90 ± 4.23	8.30 ± 0.21	67.44 ± 4.55	2.7 ± 0.90	5.03 ± 0.33
8	82.5	8.75	8.75	16.45 ± 6.02	9.50 ± 0.73	65.89 ± 2.67	3.12 ± 0.60	4.9 ± 0.62
9	90	5	5	14.06 ± 2.34	8.10 ± 0.32	70.43 ± 6.88	2.2 ± 0.77	5.4 ± 0.49

**(b):** 

Factors			Responses							
PRF (g)	SP (g)	CNF (g)	Fe (mg)	Ca (mg)	Na (mg)	Zn (mg)	Mg (mg)	K (mg)	Pho (mg)	Caro (mg)
75	16.25	8.75	18.84 ± 1.45	660.56 ± 43.78	150.80 ± 56.45	5.3 ± 0.23	44.79 ± 6.43	600.00 ± 23.73	410 ± 34.56	16.49 ± 0.40
85	5	10	15.37 ± 2.87	589.47 ± 84.65	120.87 ± 78.45	4.4 ± 0.54	72.41 ± 3.23	572.50 ± 45.21	285.95 ± 23.45	7.20 ± 0.72
75	20	5	19.97 ± 0.40	623.20 ± 12.45	138.90 ± 44.21	5.4 ± 0.03	69.00 ± 3.56	636.30 ± 12.87	352.85 ± 82.01	18.31 ± 0.89
80	12.5	7.5	17.84 ± 5.56	699.80 ± 73.53	130.08 ± 78.29	4.8 ± 0.02	43.50 ± 3.45	591.24 ± 56.76	347.28 ± 61.02	12.18 ± 0.42
77.5	16.25	6.25	20.62 ± 1.78	682.50 ± 0.32	141.70 ± 54.11	5.0 ± 0.21	48.00 ± 6.34	625.62 ± 43.22	400.63 ± 29.88	13.35 ± 0.10
70	20	10	21.56 ± 1.22	538.80 ± 53.96	152.00 ± 73.45	5.8 ± 0.88	32.00 ± 0.23	645.62 ± 39.23	397.80 ± 45.34	24.04 ± 0.21
85	8.75	6.25	16.53 ± 5.04	650.80 ± 86.88	117.62 ± 0.46	4.2 ± 0.67	44.45 ± 0.55	586.93 ± 94.03	280.32 ± 67.56	8.93 ± 0.30
82.5	8.75	8.75	17.19 ± 2.73	666.00 ± 49.40	136.40 ± 0.56	4.6 ± 0.22	62.25 ± 0.23	568.21 ± 45.65	320 ± 56.34	9.93 ± 0.70
90	5	5	13.8 ± 8.34	508.85 ± 12.45	81.99 ± 0.43	3.5 ± 0.45	32.00 ± 0.23	550.17 ± 12.62	224.642 ± 3.65	8.63 ± 0.51

PRF: parboiled rice flour; SP: spirulina powder; CNF: cashew nut flour; P: proteins; L: lipids; C: carbohydrates; A: ash; F: fibers; Fe: iron; Ca: calcium; Na: sodium; Zn: zinc; Mg: magnesium; Pho: phosphorous; K: potassium; Caro: carotenoids.

**Table 3:** Coefficient of determination (*R*^2^), AAMD, and Bf of the different responses.

Responses	*R* ^2^ (%)	AAMD	Bf
Proteins (g)	99.92	0	1
Lipids (g)	99.88	0	1
Carbohydrates (g)	99.83	0	0.9
Ash (g)	99.86	0	0.9
Fibers (g)	99.01	0	1
Iron (mg)	93.79	0	0.9
Calcium (mg)	99.95	0	1
Sodium (mg)	97.56	0	0.9
Zinc (mg)	99.86	0	0.8
Magnesium (mg)	95.06	0	0.9
Potassium (mg)	91.21	0	0.9
Phosphorus (mg)	93.74	0	1
Carotenoids (mg)	99.05	0	1
Standard values	>75%	0	0.75 < Bf < 1.25

**Table 4:** Optimal composition of the formulated weaning flour.

Responses	Optimal predicted values	Optimal experimental values	Desirability
Proteins (g)	15.00^a^	14.02 ± 0.01^a^	0.99
Lipids (g)	9.59^a^	9.04 ± 0.77^a^	1.00
Carbohydrates (g)	67.18^a^	68.48 ± 0.01^a^	1.00
Ash (g)	2.96^a^	3.00 ± 0.10^a^	0.96
Fibers (g)	5.07^a^	4.98 ± 0.50^a^	1.00
Iron (mg)	15.37^a^	16 ± 0.62^a^	0.92
Calcium (mg)	592.9^a^	510 ± 0.59^b^	1.00
Sodium (mg)	121.7^a^	123 ± 0.09^a^	1.00
Zinc (mg)	4.22^a^	5.00 ± 1.25^a^	1.00
Magnesium (mg)	63.3^a^	65 ± 0.75^b^	1.00
Potassium (mg)	568.3^a^	581.8 ± 0.11^b^	1.00
Phosphorus (mg)	273.4^a^	282.5 ± 0.46^b^	1.00
Carotenoids (mg)	7.93^a^	8.2 ± 0.95^a^	0.93
Composite desirability: 0.98			

Means ± standard deviations followed by the same letter in the same column indicate that the differences are not significant (*p* ≤ 0.05).

**Table 5:** Nutritional composition of the optimal infant flour.

Parameters	Values
Proteins (g)	14.02 ± 0.02
Lipids (g)	9.04 ± 0.77
Carbohydrates (g)	68.48 ± 0.01
Fibers (g)	4.98 ± 0.50
Calcium (mg)	510 ± 0.59
Iron (mg)	16.00 ± 0.62
Zinc (mg)	5.00 ± 1.25
Potassium (mg)	581.8 ± 0.11
Magnesium (mg)	63.25 ± 0.75
Sodium (mg)	123 ± 0.09
Phosphorus (mg)	282.5 ± 0.46
Carotenoids (mg)	8.2 ± 0.95
Energy (kcal)	411.36 ± 0.67

**Table 6:** Pasting properties of optimized flour.

Parameters	Values
Pasting temperature (°C)	74.25 ± 1.42
Peak viscosity (cP)	54 ± 0.54
Holding viscosity (cP)	0
Breakdown (cP)	0
Final viscosity (cP)	86 ± 0.70
Setback (cP)	26 ± 0.70

## Data Availability

The datasets used and/or analyzed during the current study are available from the corresponding author on reasonable request.
